# Clinical Characteristics of Children With Celiac Disease Not Responding to Hepatitis B Vaccination in India

**DOI:** 10.1097/PG9.0000000000000046

**Published:** 2021-01-13

**Authors:** Aradhana Aneja, Sadhna B. Lal, Arun K Sharma, Amit Rawat, Surjit Singh

**Affiliations:** From the * Division of Paediatric Gastroenterology, Hepatology & Nutrition,; † Section of Virology, Department of Gastroenterology,; ‡ Division of Paediatric Immunology, Post Graduate Institute of Medical Education & Research, Chandigarh, India.

**Keywords:** immune response, hepatitis B vaccine, children, celiac disease

## Abstract

The immunological response to hepatitis B virus (HBV) vaccine may be suboptimal in children with celiac disease (CD), but the reasons for this are not well defined.

**Objectives:**

This study was undertaken to assess the immune response to HBV vaccine in CD children and to explore the possible factors affecting the immune response.

**Methods:**

The study population consisted of 3 groups—50 newly diagnosed CD children (group 1), 50 previously diagnosed CD children who were on gluten free diet (GFD) >3 months (group 2), and 100 age and gender matched healthy controls (group 3). The patient characteristics were recorded, and the blood samples were analyzed for HBsAg and anti-HBs titers. The nonresponders were given a booster dose of HBV vaccine and reevaluated after 6 weeks.

**Results:**

Positive anti-HBs response was found in 46% in newly diagnosed CD children, 60% in CD children on GFD, and 83% in healthy controls (*P* < 0.001). The immune response to HBV vaccine in CD children was inferior to that in healthy children (53% vs 83%, *P* < 0.001). The immune response was found to be significantly affected by age at diagnosis, delay in diagnosis, type of presentation, and compliance to GFD. 44 out of 45 (97.77%) nonresponders from CD group seroconverted after a single booster dose.

**Conclusion:**

Early diagnosis and good compliance to GFD may improve the immune response to HBV vaccine in CD children. Single additional booster dose is sufficient to attain optimal immune response.

**What Is Known**

- Children with celiac disease may have a suboptimal response to Hepatitis B vaccine
- The ongoing gluten intake might affect the immune response

**What Is New**

- Poor response to hepatitis B virus vaccine is significantly associated with age at diagnosis, delay in diagnosis, type of presentation, and compliance to gluten-free diet.
- Ongoing gluten intake has significant impact on protective immune response to Hepatitis B vaccine

**Translational Impact**

- Evaluation of immune response to Hepatitis B vaccine should be considered as part of routine assessment in children with celiac disease
- Strict compliance to gluten-free diet and an extra booster dose is required to have a protective immune response to Hepatitis B Vaccine

**H**epatitis B virus (HBV) infection is a major global health problem with significant morbidity and mortality (1). Universal HBV vaccination has dramatically reduced the incidence of HBV infection in children (1–3). Primary immunization induces a protective humoral response to HBV vaccine in >90% of children, which however declines with time (2–6).

Celiac disease (CD) is a gluten-dependent immunologically mediated systemic disorder in genetically susceptible individuals, characterized by a variable combination of clinical manifestations, CD-specific antibodies, HLA DQ2 or HLA DQ8 haplotypes, and enteropathy (7). The global prevalence of CD is 0.7%–1.4% and around 1% in the general population from Northern India (8–11). These children may be predisposed to various infections due to the malabsorptive state and hyposplenism (12–15).

India lies in the intermediate HBV endemicity zone (prevalence 2%–8%), with a higher prevalence of HBV infection reported in children as compared to adults (16). A few studies have been done to evaluate the antibody response to HBV vaccine in CD children. These have shown that CD patients have a decreased response rate (30–50%) to HBV vaccine as compared to the general population (17–23). This unresponsiveness to HBV vaccine in children with CD could make them susceptible to HBV infection and pose a public health threat. However, the clinical factors causing the lack of the immune response to HBV vaccine remain largely unexplored (17). There is some evidence that gluten intake may affect the response to HBV vaccine, but the role of duration of gluten exposure and the influence of ongoing gluten intake (possibly affected by age at diagnosis, delay in diagnosis, and compliance) has not been studied. The disease activity in CD children may cause a defective or insufficient HBsAg-specific T helper cell response, resulting in primary failure of development of protective immune response. Thus, the severity of symptoms and malnutrition caused by CD may have an adverse impact on the immune response to HBV. The literature on impact of clinical presentation and extent of malnutrition, on the immune response, is controversial (23–26). The current study was conceived to investigate the immune response to HBV vaccine and the various clinical factors influencing it in Indian children with CD.

## OBJECTIVES

The aim of this study was to

➢ Assess the response to HBV vaccine in children with CD, and compare it with age and gender matched healthy children.
➢ Investigate the various clinical factors, for example, role of duration of gluten intake, ongoing gluten intake, clinical presentation, and nutritional status that may influence the vaccine-induced immune response.
➢ Evaluate the response to a single-booster dose of HBV vaccine in nonresponders.

## PATIENTS AND METHODS

This was a cross-sectional observational study done in a tertiary care hospital of North India, including 3 study groups:

➢ Group 1: 50 newly diagnosed CD children, who were not on gluten free diet (GFD) (those who were not previously diagnosed and were never started on GFD).
➢ Group 2: 50 previously diagnosed CD children who were on GFD >3 months duration.
➢ Group 3: 100 age and gender matched healthy controls.

The data were collected from July 2018 to June 2019. All the enrolled children had received the primary immunization against HBV (as confirmed from the immunization cards) during the first 6 months of life. The primary vaccination was with recombinant HBV vaccine at 0, 1, and 6 months of life, in dose of 10 µg and was administered intramuscularly, as per national immunization protocol (27). The various pertinent details (enumerated below) were recorded. The diagnostic criteria of diagnosis of CD were based on the existing guidelines (7, 28). Patients and parents who gave a written assent and consent were included in the study. The compliance to GFD was assessed in each participant by an experienced clinical nutritionist and serum tissue transglutaminase IgA (TGA IgA) levels.

### Inclusion Criteria

➢ Children diagnosed as CD (by serology and histology)
➢ Age between 2 and 10 years
➢ Completed primary immunization against HBV, with 3 intramuscular doses (0, 1, 6-mo schedule).
➢ HBV vaccination should have been completed at least 6 months before enrollment.

### Exclusion Criteria

➢ Children with uncertain diagnosis of CD
➢ Children with incomplete/partial immunization
➢ Immunization status not confirmed
➢ Children on immunosuppression or with other comorbidities like diabetes, chronic kidney disease, chronic liver disease, and cardiovascular disorders.

### Methodology

#### Patients

The patients with CD who fulfilled the inclusion criteria and healthy controls were enrolled from the outpatient department.

### Clinical Data

The initial clinical presentation, age of onset of clinical symptoms, age of diagnosis of CD, delay in diagnosis, anthropometry, clinical examination findings, and the details of the investigations used for diagnosis of CD-like serology and histology were recorded.

### Serology

➢ 3 mL of blood was collected in plain sterile container, serum separated, and stored at −20^○^C.
➢ These samples were tested for HBsAg and anti-HBs levels by ELISA using standard ELISA kits, and the titers were reported in milli international units per microliter (mIU/µL).

### Definition of Response

➢ Positive immune response was defined by anti-HBs ≥10 mIU/µl (5).
➢ Patients with an anti-HBs titer <10 mIU/µl were defined as nonresponders (5^)^.
➢ Patients with titers between 10 and 100 mIU/µl were defined as low responders.
➢ Patients with titers >100 mIU/µl were defined as high responders.

### Booster Dose

➢ The nonresponder CD patients and nonresponder healthy controls were given an intramuscular booster dose of HBV vaccine (10 µg of recombinant HBV vaccine; Bharat Biotech International Limited, Hyderabad, India).
➢ Repeat estimation of anti-HBs (quantitative) was done between 4 and 6 weeks after booster dose administration.
➢ The patients were then reclassified into nonresponders, low responders, and high responders.
➢ The patients who were still nonresponders (after booster) were advised a repeat complete course of 3 doses of HBV vaccine at 0, 1, and 6-month intervals.

The study was approved by the Institutional Ethics Committee (NK/4773/DM/188 dated December 21, 2018).

### Statistical Analysis

Descriptive statistics were used to describe baseline variables. Quantitative variables were reported as mean with SD or median with interquartile range (depending on distribution of data), while qualitative variables were described in proportions. The difference between continuous variables was analyzed using “Mann-Whitney *U* test” if not normally distributed and student’s *t* test if normally distributed. The difference between categorical variables was analyzed using Fisher’s exact test. The “chi-square test” was used to compare whether there was significant difference in proportions of protective anti-HBs titers between different groups. If the parameters were normally distributed in all 3 groups, their group mean was compared by ANOVA followed by post hoc test for pair-wise comparison, else their distribution was compared using Kruskal-Wallis test followed by Mann-Whitney test for pair-wise comparison. A *P* value less than 0.05 was considered statistical significant. Univariate and multivariate logistic regression analysis was conducted to identify independent predictors of response. Data analysis was done on SPSS statistical software (version 22, IBM SPSS Inc. Chicago, IL).

## RESULTS

Fifty consecutive CD children meeting the inclusion criteria were enrolled in each group (1 and 2). One hundred age- and gender-matched healthy controls were enrolled in group 3.

### Baseline Characteristics

The median age of newly diagnosed CD children, CD children on GFD, and healthy controls was 7, 7.2, and 7 years, respectively (*P* = 0.389) (Table 1). There was a female preponderance in the CD children, although male preponderance was seen in the healthy controls (62 male and 38 female children). Among the CD children (n=100), infantile onset (onset ≤2 yrs of age) was seen in 31% of patients and the median age at diagnosis was 5 years (3–7 yrs). The median delay in diagnosis was calculated from the time of onset of symptoms to initiation of GFD. The median delay in diagnosis was 1.0 yr (0.2–2 yrs), and the median delay in newly diagnosed CD children was significantly higher as compared to CD children on GFD (2 yrs vs 0.5 yrs, *P* = 0.023). The most common clinical feature was short stature, seen in 72% of patients, followed by anemia in 59% of children. Among the gastrointestinal manifestations, abdominal pain was seen in 61% of children, diarrhea and abdominal distension (51% each) and constipation in 33% children. On comparing the growth parameters taken at the time of enrollment, the median height for age was significantly lower in the newly diagnosed CD children as compared to CD children on GFD (−2.09 vs −1.22; *P* = 0.003). On comparison of the growth parameters between compliant and noncompliant CD children, it was found that the median height for age was significantly lower in the noncompliant CD children as compared to compliant CD children (−1.74 vs −1.0, *P* = 0.047).

**Table 1.** Clinical Characteristics Between Newly Diagnosed CD Children and CD Children on GFD

Variable	Newly Diagnosed CD Children, No (%)	CD Children on GFD, No (%)	*P*
Gender			
Female	24 (48%)	28 (56%)	0.423
Male	26 (52%)	22 (44%)	
Median age of onset (yr)	5 (IQR, 2–6)	3 (IQR, 2–5)	**0.009**
Median age at diagnosis (yrs)	7 (IQR, 5–9)	4 (IQR, 3–6)	**0.001**
Median delay in diagnosis (yrs)	2.0 (IQR, 0.2–3)	0.5 (IQR, 0.17–1.62)	**0.023**
Presentation			
Classical	20 (40%)	34 (68%)	**0.005**
Non classical	30 (60%)	16 (32%)	
Wt for Ht z score	−1.27 (IQR, −1.88 to −0.44)	−0.67 (IQR, −1.8 to −0.04)	0.087
Ht for age z score	−2.09 (IQR, −2.84 to −1.35)	−1.22 (IQR, −2.28 to −0.28)	**0.003**
TGA IgA >10 times	49 (98)	42 (84)	0.076
Histology			
Marsh 2	6 (12)	5 (10)	0.683
Marsh 3a	10 (20)	12 (24)	
Marsh 3b	33 (66)	32 (64)	
Marsh 3c	1 (2)	0 (0)	

CD = celiac disease; GFD = gluten free diet.

### GFD Duration and Compliance

The median duration of GFD intake in CD children on GFD was 2 years (1–4.2 yrs). The compliance was assessed by dietary review, and it was correlated with follow-up TGA IgA values, which were available for 44 patients. It was found that out of total 50 patients in CD children on GFD, 66% (33) of patients had good compliance and 34% (17) of patients had poor compliance as per dietary review. It was found that TGA IgA negativity was seen in 93% of patients with good dietary compliance as compared to 56% of patients with poor dietary compliance (*P* = 0.002).

### HBs Ag and Anti-HBs Response

The HBsAg was negative for all CD children as well as healthy controls (Table 2). Positive anti-HBs response was found in 46% (23) of newly diagnosed CD children, 60% (30) in CD children on GFD, and 83% (83) in healthy controls (*P* < 0.001). The CD children (both newly diagnosed and those on GFD) were either nonresponders (47%) or low responders (33%), whereas in healthy controls, 38% of children were high responders (*P* < 0.001). The number of children with positive anti-HBs response was higher in CD children on GFD as compared to newly diagnosed CD children, but the difference was not statistically significant (*P* = 0.161).

**Table 2.** Comparison of Grade of Anti-HBs Titers in 3 Groups

Anti HBs Titer (mIU/µl)	Newly Diagnosed CD Children	CD Children on GFD	Healthy Controls	*P*
<10	27 (54%)	20 (40%)	17 (17%)	**<0.001**
10–100	14 (28%)	19 (38%)	45 (45%)	0.130
>100	9 (18%)	11 (22%)	38 (38%)	**0.018**
Median anti-HBs titer	70 (11–280)	55 (14–170)	88 (15–590)	0.146

CD = celiac disease; GFD = gluten free diet.

### Comparison Between Responder and Nonresponder CD Patients

Univariate analysis between the responders and nonresponders among CD patients revealed that age at diagnosis, delay in diagnosis, and compliance to GFD were the factors, which were statistically different between the responders and nonresponders. Age at diagnosis of responders was significantly lower as compared to nonresponders (5 vs 7 yrs, *P* = 0.002). The delay in diagnosis was significantly longer for the nonresponders as compared to the responders (2 vs 0.3 yrs, *P* < 0.001). The compliant CD children had a better response as compared to the noncompliant CD children (84.8% vs 11.8%, *P* < 0.001).

In the multivariate analysis, age at diagnosis with an OR of 0.576 (95% CI: 0.443-0.749, *P* < 0.001) and delay in diagnosis with an OR of 0.483 (95% CI: 0.303-0.770, *P* = 0.002) correlated with poor response whereas the absence of diarrhea (nonclassical presentation) with an OR of 5.344 was associated with better anti-HBs response (95% CI: 1.617-17.66, *P* = 0.006).

### Correlation of HBV Vaccine Response in Newly Diagnosed, Compliant, and Noncompliant CD Children

A comparison of newly diagnosed, compliant, and noncompliant CD children was done (Table 3). It was found that majority of the newly diagnosed CD children and the noncompliant CD children had negative anti-HBs response (54% and 88%, respectively) as against the compliant CD children where majority (85%) had a positive anti-HBs response (*P* < 0.001). On comparison of newly diagnosed CD children with delay >2 years (median delay) with noncompliant CD children, it was found that both the groups were similar and majority of the patients in both the groups had negative anti-HBs response (64% vs 89%, *P* = 0.079).

**Table 3.** Correlation of Anti-HBs Response With Gluten Exposure

Anti-HBs Response	Newly Diagnosed CD Children	CD Children With Good Compliance to GFD	CD Children With Poor Compliance to GFD	*P*
Positive	23 (46%)	28 (84.8%)	2 (11.8%)	**<0.001**
Negative	27 (54%)	5 (15.2%)	15 (88.2%)	**<0.001**

CD = celiac disease; GFD = gluten free diet.

### Post Booster Response

The nonresponders were given a booster dose of HBV vaccine (10 µg/mL IM), and anti-HBs response was reevaluated. In newly diagnosed CD children, all 27 nonresponder patients received a booster dose of Hepatitis B vaccine, but 2 did not come for repeat anti-HBs evaluation. Out of 25 patients for whom repeat anti-HBs evaluation was done, 24 patients (96%) seroconverted, but 1 patient did not seroconvert even after the booster dose. In CD children on GFD, all 20 patients received a booster dose of hepatitis B vaccine and were also reevaluated for anti-HBs response. All 20 patients seroconverted after the booster dose. The median anti-HBs value postbooster was 440 mIU/mL (240–917.5) in newly diagnosed CD children and 675 mIU/mL (63.75–952.5) in CD children on GFD. In healthy controls, out of 17 nonresponders, 8 had come for the booster dose. All 8 children seroconverted after the booster dose and showed a high response.

## DISCUSSION

Various studies have found that children with CD have a suboptimal response to HBV vaccine (17–23), but none has evaluated the role of clinical factors affecting the immune response. In our study, positive anti-HBs response was significantly lower in CD children as compared to controls (53% vs 83%). The possible confounding factor of waning of immune response with age was eliminated, as the age of children in both groups was similar. This is similar to the results found by Leonardi et al (21), where half of CD children were nonresponders, as against 12% of the controls.

At baseline, the age distribution was comparable in all the 3 groups. The median age of onset, the median age of diagnosis, and the delay in diagnosis was significantly higher in the newly diagnosed CD children as compared to the CD children on GFD. The most common clinical presentation was short stature, and as expected, stunting was significantly more common in the newly diagnosed children as compared to the CD children on GFD. Similar results have been earlier described from India, where the short stature was seen in 60–100% of CD children (29–32).

The anthropometry was recorded at the time of enrollment, and the newly diagnosed CD children were found to be significantly stunted, as compared to CD children on GFD. This difference can be explained by ongoing gluten consumption in the newly diagnosed CD children. We also found that the median height for age z score was significantly lower in the noncompliant CD children as compared to CD children with good compliance. Thus ongoing gluten intake has a significant impact on the growth of CD children.

The mechanism for poor anti-HBs response in CD patients is not clear. CD being strongly associated with HLA DQ2, B8, and DR3, this may be an explanation for a suboptimal anti-HBs response, as previously suggested (33–35). Intestinal injury in CD results from the interaction of deamidated glutamine residues of gliadin with HLA-DQ2/DQ8 molecules. The failure of the development of protective anti-HBs response may be due to HBsAg and gliadin peptides competing with each other to bind to HLA-DQ2 (36, 37).

The newly diagnosed CD children had lower response rate as compared to those already on GFD (46% vs 60%), although the difference was not statistically significant. In CD children on GFD, the patients were checked for compliance and we evaluated the correlation of anti-HBs response and GFD compliance. We had considered that patients who were on strict home-based GFD and not consuming marketed products to be well compliant because various studies have done earlier (38–40) have shown that gluten contamination is frequently reported in labeled commercially available GFD products. This is especially so in India, where mandatory checking of commercial GFD for gluten contamination is not enforced by law. This fact was confirmed by the good correlation between TGA positivity and poor compliance on dietary assessment in this study.

It was found that patients who were compliant to GFD were more likely to have positive anti-HBs values than the noncompliant patients (84.8% vs 11.8%). Also, on comparison between newly diagnosed CD children, compliant CD children, and noncompliant CD children, it was found that newly diagnosed CD children and noncompliant CD children had significantly higher negative anti-HBs response. Further, analysis of newly diagnosed children with longer delay (>2 yrs) and noncompliant CD children revealed that both the groups were similar and majority had a negative anti-HBs response. The newly diagnosed and the noncompliant CD children have ongoing gluten exposure, and this ongoing gluten intake interferes with the development of HBsAg specific T helper response, leading to low immune response. As both the healthy controls and the CD children had received HBV vaccination at first 6 months of life, the significant difference in immune response clearly indicates the influence of ongoing gluten intake. In CD children, the interaction between specific-deamidated glutamine residues of gliadin and HLA DQ2/HLA DQ8 induces proliferation of T lymphocytes. On HBV vaccination, both HBsAg protein fragments and gliadin peptides bind to HLA DQ2 molecules and their competition results in defective protective immune response. Thus, compliance to GFD has a significant impact on the development of positive anti-HBs response.

Nemes et al (17) have previously shown that reimmunization with HBV vaccine produced protective anti-HBs response in 95% of CD children who were on a GFD, as against 51% of CD children who were not on a GFD. However, a recent study by Hweta et al, showed conflicting results where the sero response rate in children with CD on GFD was similar to healthy controls (41). This study included CD children with a mean age of 8 yrs, which was not matched with the age of the controls, which was 5 yrs. Thus, both controls and study groups were not comparable and valid conclusions cannot be drawn from this study.

On the evaluation of postbooster response in our study, seroconversion was seen in 96% in newly diagnosed CD children and 100% in CD children on GFD and healthy controls. Thus, the response to the booster dose of HBV vaccine in CD children who were on GFD was similar to healthy children. The CD children were either nonresponders or low responders initially but were converted to high responders after the booster dose. This reflects the anamnestic anti-HBs response after the booster dose, and thus, the intact immunological memory in these patients (37).

Both univariate and multivariate analysis showed that children with a longer delay in diagnosis (from the onset of symptoms to institution of GFD) were more likely to be nonresponders. This finding has not been emphasized earlier and indicates the role of ongoing gluten intake in attenuating the immune response.

We also found on multivariate analysis that children with no diarrhea were likely to have a better antibody response as compared with those having diarrhea as a presenting feature. Although the height and weight in the children with diarrhea were similar to those without diarrhea, it is possible that children with diarrhea and malabsorption have more nutritional deficiencies accounting for worse immunologic response to the vaccine.

Another question that needs to be answered is whether the CD children need evaluation of anti-HBs levels as part of the baseline workup and whether the nonresponders need revaccination. India has a high prevalence of CD (1%) and intermediate endemicity for HBV (8–10, 16). Previous studies have shown that the prevalence of HBs-Ag positivity in CD is higher than in the general population (42).

Our study demonstrates that immune response to HBV vaccine in CD children is suboptimal, as has been shown by few previous studies (17, 20, 21). The literature suggests that that the nonresponder children are unprotected and are at higher risk of acquiring HBV infection than those who are poor responders (5^)^. Few studies have also found HBsAg positivity in subjects who had completed the primary HBV vaccination schedule (43, 44). Thus, it is recommended that nonresponders should be administered additional vaccines until they achieve sero protective levels (5, 45).

A limitation of our study is that compliance was checked by standard dietary review and TGA only. There is no evidence that these methods may not be foolproof and do not correlate well with histologic recovery (46). However, our study is unique in that we analyzed 3 groups, of which group 1 included newly diagnosed CD children. This has not been previously assessed.

In conclusion, our study is the first study from India to assess the immune response to HBV vaccine in children diagnosed with CD. Our study confirms the suboptimal response to HBV vaccine in children with CD. We also analyzed the various clinical factors associated with the poor response of HBV vaccine in CD children and found that age at diagnosis, delay in diagnosis and institution of treatment, GFD compliance, and type of CD presentation (diarrhea vs no diarrhea) were independent predictors of immune response to HBV vaccine. Of these, the role of delayed institution of treatment has been highlighted for the first time. We recommend that anti-HBs evaluation should be considered at the time of diagnosis of CD, giving clinicians an opportunity to protect these children with an additional booster dose of HBV vaccine.
